# Identification of a metagenomic gene cluster containing a new class A beta-lactamase and toxin-antitoxin systems

**DOI:** 10.1002/mbo3.104

**Published:** 2013-07-22

**Authors:** Ken Vercammen, Tamara Garcia-Armisen, Nathalie Goeders, Laurence Melderen, Josselin Bodilis, Pierre Cornelis

**Affiliations:** 1 Department of Bioengineering Sciences Research group Microbiology and VIB Department of Structural Biology, Vrije Universiteit BrusselPleinlaan 2, 1050, Brussels, Belgium; 2 Ecologie des Systèmes Aquatiques, Université Libre de Bruxelles, Campus de la PlaineCP 221 Boulevard du Triomphe, 1050, Brussels, Belgium; 3 Génétique et Physiologie Bactérienne, Université Libre de Bruxelles, IBMM-DBM12 rue des Professeurs Jeneer et Brachet, 6041, Gosselies, Belgium; 4 Microbiologie Environnementale et Biologie Évolutive UFR Sciences et Techniques, Université de RouenPlace Emile Blondel, 76821, Mont-St-Aignan, France

**Keywords:** Antibiotic resistance, metagenomic DNA, toxin–antitoxin systems, β-lactamase

## Abstract

Several reports mention the presence of antibiotic resistance genes in natural and polluted environments, but many studies are based on their detection via polymerase chain reaction (PCR amplification of known genes and not on an activity screening. We constructed a metagenomic fosmid bank from DNA isolated from a polluted river in Brussels, Belgium, the Zenne. A total of 120,000 clones were pooled and plated directly on solid media containing different antibiotics. Several clones were isolated which could grow in the presence of ampicillin. The DNA from several clones was extracted and subjected to restriction analysis and, based on their restriction pattern, two different clones were found. One of the clones was selected for further study as it showed a higher level of resistance to different β-lactams antibiotics (ticarcilline and ceftazidime). To find out which gene is responsible for the resistance, an in vitro transposon mutagenesis was performed and clones having lost the resistance phenotype were analyzed via inverse PCR amplification. Several clones had an insert in a gene encoding a new type of β-lactamase. The amplified fosmid DNA was fully sequenced revealing an insert of 41 kb containing 39 open reading frames (ORFs). Transposon insertions inactivating the resistance to β-lactams were also found in the ORF upstream of the *blaA* gene, encoding an aminotransferase, suggesting a polar effect on the transcription of the gene downstream. In addition, other genes were found such as histidine biosynthesis genes, which were found to be scattered on the insert, a *relA*/*spoT* gene, and genes belonging to type II toxin–antitoxin system. This predicted system was experimentally validated in *Escherichia coli* using an inducible expression system.

## Introduction

It is now well established that natural environments can be a reservoir of antibiotic resistance genes (Martinez et al. 2009; Allen et al. 2010). The increased use of antibiotics in agriculture and in medicine has also caused a massive release of antibiotics in streams and rivers, allowing the selection of antibiotic resistant bacteria (Baquero et al. 2008; Martinez et al. 2009; Allen et al. 2010). As a result of industrial activity, toxic metals are also released in streams selecting for bacteria able to resist both metal and antibiotic imposed stresses (Calomiris et al. 1984; Baker-Austin et al. 2006). A recent study revealed a positive correlation between the levels of metals and resistance genes (Knapp et al. 2011). For metals, as well as for antibiotics, efflux via efflux pumps is involved in resistance (Grass et al. 2011; Kim et al. 2011). Some metals such as copper or chromium can cause an important production of reactive oxygen species (ROS) via a Fenton reaction (Lloyd and Phillips 1999), and recently, it has been proposed that many antibiotics also generate an oxidative stress (Kohanski et al. 2010; Martinez and Rojo 2011). Metal and antibiotic resistance genes can be moved between bacteria via horizontal transfer of mobile genetic elements (de la Cruz and Davies 2000; Gootz 2010; Andam et al. 2011; Zhang et al. 2011). Two approaches can be used to detect antibiotic resistance genes from metagenomic DNA. The first one is based on the amplification of known antibiotic resistance genes using gene-specific markers, whereas the second, termed functional metagenomics, involves the construction of metagenomic libraries, which are further screened for expression of drug resistance (Riesenfeld et al. 2004; Allen et al. 2010). With the power of new sequencing methods, it is also possible to sequence the inserts of metagenomic clones, hence discovering the genomic context. In this study, we present the construction of a polluted stream metagenomic library, which was functionally screened for beta-lactam resistance, and the identification of a new beta-lactamase gene as well as its genomic context. We also present evidence for the presence and biological activity of toxin–antitoxin genes.

## Materials and Methods

### Strains and plasmid constructions

See Table 1 for the list of used strains and plasmids.

**Table 1:** Strains and plasmids used for this study

Strain or plasmid	Genotype or plasmid properties	Reference or source
*Yeast*		
23344c	MAT ura3	Our laboratory
*Bacterial strains*		
MC1061	*araD*139,D(*lacI* POZY)74, *galU galK rpsL*, Δ(*araA* BC-*leu*)7697, *hsdR mcrB*, Sm^s^	Casadaban and Cohen (1980)
MG1655	Wild-type *E. coli* K-12	Xiao et al. (1991)
DJ624 Δara	MG1655 Δ*lac malP*::*lacI*q	Our laboratory
*Plasmids*		
pBAD33	P15A, CamR, pBAD promoter	Guzman et al. (1995)
pBAD33lev	pBAD33lev derivative containing URA3 selection gene and a yeast replication origin	This work
pBADlev-relE93	pBAD33lev derivative containing the gene relE_93_ under the pBAD promoter	This work
pBADlev-ORF24	pBAD33lev derivative containing the ORF24 under the pBAD promoter	This work
pBADlev-ORF26	pBAD33lev derivative containing the ORF26 under the pBAD promoter	This work
pKK223-3lev	ColE1, AmpR, pTac promoter	Brosius and Holy (1984)
pKK223-3lev-relB93	pKK223-3lev derivative containing the *relB*_93_ gene under the control of the pTac promoter	This work
pKK223-3lev-ORF25	pKK223-3lev derivative containing ORF25 under the control of the pTac promoter	This work
pKK223-3lev-ORF27	pKK223-3lev derivative containing ORF27 under the control of the pTac promoter	This work

### Media and growth conditions

Yeasts were grown on 863 media (1% yeast extract, 1% bactopeptone, and 2% glucose). Luria-Bertani medium (LB) and M9 minimal medium (KH_2_PO_4_ (22 mmol/L), Na_2_HPO_4_ (42 mmol/L), NH_4_Cl (19 mmol/L), MgSO_4_ (1 mmol/L), CaCl_2_ (0.1 mmol/L), NaCl (9 mmol/L), vitamin B1 (1 μg/mL)) supplemented with casamino acids and carbon sources were used to grow bacteria. Ampicillin and chloramphenicol were added at respective final concentration of 50 and 12.5 μg/mL. Isopropyl β-D-1-thiogalactopyranoside (IPTG) was used at 0.1 mmol/L.

### Freshwater sampling and treatment

Freshwater was collected in November 2008 and February 2009 from the surface of the Zenne river at the Buda bridge (Haren, Belgium) situated at an industrial site. The Zenne water (2 L) was transferred into sterile bottles at 4°C and immediately filtered using a 1.2-μm glass fiber filter (Whatman, GE Healthcare) to eliminate the sediments and further through a 0.2-μm filter to capture the microorganisms. The 0.2 μm filters with the retained microorganisms were stored at −80°C and thawed at room temperature just before extraction of the DNA.

### DNA extraction

DNA from the filters was extracted using the Metagenomic DNA Isolation kit for Water from Epicentre®. The final concentration of DNA was 0.27 μg/mL.

### Metagenomic library construction

Electrocompetent *Escherichia coli* TransformMax EPI300 T1R cells (Epicentre®, Madison, WI) were used for library construction and grown on LB broth at 37°C containing chloramphenicol (Cm) (12.5 μg/mL). The metagenomic library was constructed using the CopyControl Fosmid Library Production Kit from Epicentre®. Briefly, the extracted DNA was blunt ended at the 5′-PO_4_- site, ligated into the pCC1FOS vector and packaged into λ phages using MaxPlax™ Lambda packaging extracts (Epicentre®). *E. coli* EPI-300 T1R cells were infected with the packaged bacteriophages and the clones inoculated on LB plates containing 12.5 μg/mL Cm. Recombinant clones growing on Cm plates were scraped from the selective agar plates into 10 mL of LB medium plus 20% glycerol and stored in aliquoted 1 mL pools at −80°C. The final titer was 10^11^ colony-forming units (CFU) per mL.

### Control of the quality of the metagenomic library

Twenty colonies growing on selective medium plus Cm were selected randomly and grown in 1 mL LB medium plus Cm plus the Copy Control Autoinduction Solution from Epicentre. The DNA was extracted according to the manufacturer protocol (Epicentre) and was restricted using the Fast Digest Restriction enzymes *Nde*I, *Pst*I, and *Hind*III (Fermentas, Thermo Scientific).

### Functional screening of the metagenomic library

The *E. coli* TransformMax EPI300 T1R::pCC1FOS cells were tested for ampicillin susceptibility using the minimum inhibitory concentration assay (MIC, Oxoid, Thermo Scientific) and were found to be sensitive to less than 10 μg mL Amp. The metagenomic library was therefore plated on LB agar containing 12.5 μg/mL Cm and 50 μg/mL ampicillin. The ampicillin resistant clones (20 clones) were also tested for resistance to other β-lactams, including amoxicillin, aztreonam, cefepime, ceftazidime, ceftriaxone, meropenem, piperacillin, and ticarcillin, using the disk assay (each disk contains 30 μg of one antibiotic). The resistant clones were analyzed by restriction digestion using fast digest *Pst*I and *Nde*I restriction enzymes (Thermo scientific).

### In vitro transposon mutagenesis

The EZ-Tn5<KAN-2> insertion kit (Epicentre®) was used for the generation of ampicillin-sensitive clones. Briefly, the fosmid carrying the ampicillin resistance gene was purified using the Qiagen miniprep kit. First a 1:1000 diluted overnight culture was induced for 5 h at 37°C at 225 rpm with the CopyControl Autoinduction Solution (Epicentre®) in order to increase the plasmid copy number. The purified recombinant fosmid was then incubated for 2 h with the transposon carrying the kanamycin resistance gene and the transposase under the conditions recommended by the manufacturer. This mixture was used for transformation of the *E. coli* TransformMax EPI300 T1R cells. The transposon mutants were grown on LB containing Cm and LB containing Cm and Amp (50 μg/mL). The fosmids of the candidates having lost their resistance to ampicillin were extracted, as mentioned above, and sent for sequencing using the KAN-2 FP-1 forward primer (5′ ACCTACAACAAAGCTCTCATCAACC 3′) and KAN-2 RP-1 reverse primer (5′ GCAATGTAACATCAGAGATTTTGAG 3′).

## 454 pyrosequencing and gene annotation

The clone containing the multiresistant ampicillin fosmid was sent for sequencing. Briefly, the Amp^R^ clone was grown overnight at 37°C and purified as mentioned above followed by an 1-hour incubation with Riboshredder RnaseA (Epicentre). The 454 pyrosequencing reaction was done by DNAvision (Gosselies, Belgium). The sequences were assembled using CAP3 version 3 (available at Mobyle Pasteur: http://mobyle.pasteur.fr/cgi-bin/portal.py#forms::cap3) and annotated by the free online program Softberry (http://linux1.softberry.com/berry.phtml?topic=fgenesb&group=programs&subgroup=gfindb).

### Phylogenetic analysis

In order to get a broad picture of the diversity of the Class A Beta-lactamase, 29 reference sequences were chosen from the study of Hall and Barlow (2004). In addition, the five best BLAST hits were added with the new sequences identified in this study. Altogether, the 35 protein sequences were aligned with ClustalW2 with default parameters (Larkin et al. 2007) implemented in Seaview 4.2.4 (Gouy et al. 2010). After removing positions with gaps, conserved blocks of the multiple alignments were selected by using Gblocks 0.91b with default parameters (Talavera and Castresana 2007). A neighbor-joining tree was inferred using MEGA v5.0 (Tamura et al. 2011) with Poisson correction. The phylogeny was rooted with the group that includes CGA1, CME2, and CFXA2 because those genes are in the chromosomes of species within the *Cytophaga*–*Flexibacter*–*Bacteroides* (CFB) group, the group that is closest to the root of the Eubacterial tree among the groups represented in this phylogeny (Hall and Barlow 2004). The node supports (on the branches) were evaluated based on 1000 bootstrap replicates and the bar indicates sequence divergence.

### Cloning of toxin and antitoxin genes

Primers used to amplify genes are flanked by 40 bp homologous to the cloning sites of the pBAD33-lev or the pKK223-3-lev vectors. These pBAD33 (Guzman et al. 1995) and pKK223-3 vectors contain the URA3 selection gene and a yeast replication origin obtained from the pFL44 vector (Brosius and Holy 1984; Bonneaud et al. 1991) (see Table 2). Polymerase chain reaction (PCR) products were cotransformed with the linearized pBAD33-lev or pKK223-3-lev vectors into yeast by Gietz and Schiestl (2007) method. Plasmids were constructed by in vivo recombination between PCR products and linearized plasmids in yeast. Candidate vectors were sequenced and transformed into the *E. coli* MC 1061 strain and then into the DJ624 Δ*ara*.

**Table 2:** Oligonucleotides used in this study, 40 bp regions homologous to the pBAD33lev and pKK223-3lev required for in vivo recombination yeast are underlined

Primer	Sequence (5′-3′)
ORF24-for-Bad-lev-xba1	TTTTTGGGCTAGCGAATTCGAGCTCGGTACCCGGGGATCCTCTAGA**AGGAGGgagta**ATGAAAAATGAAATAATCGAC
ORF24-rev-Bad-lev-pst1	AAATCTTCTCTCATCCGCCAAAACAGCCAAGCTTGCATGCCTGCAGTTATTTAATCGCATCACTGAG
ORF25-for-pkk-lev-ecoR1	TGTGGAATTGTGAGCGGATAACAATTTCACACAGGAAACAGAATTCATGAAAACTAAAGCACTTTTTG
ORF25-rev-pkk-lev-pst1	CTGAAAATCTTCTCTCATCCGCCAAAACAGCCAAGCTTGGCTGCAGTCATTGTAAATTGTATTCTTC
ORF26-for-Bad-lev-xba1	TTTTTGGGCTAGCGAATTCGAGCTCGGTACCCGGGGATCCTCTAGA**AGGAGGgagta**ATGGCAAATTTAGAACCATTC
ORF26-rev-Bad-lev-pst1	AAATCTTCTCTCATCCGCCAAAACAGCCAAGCTTGCATGCCTGCAGTTATCTTCTACCTCTTTGACC
ORF27-for-pkk-lev-ecoR1	TGTGGAATTGTGAGCGGATAACAATTTCACACAGGAAACAGAATTCATGTCAAAATGGATTGATAA
ORF27-rev-pkk-lev-pst1	CTGAAAATCTTCTCTCATCCGCCAAAACAGCCAAGCTTGGCTGCAGCTATTTATCGTTGTTGCTC
relB93-for-lev-ecoR1	TGTGGAATTGTGAGCGGATAACAATTTCACACAGGAAACAGAATTCATGAAAACAGAAAACCCCGT
relB93-rev-lev-pst1	CTGAAAATCTTCTCTCATCCGCCAAAACAGCCAAGCTTGGCTGCAGTTAGAGCCATTTGGTTAAAAC
relE93-for-lev-xba1	TTTTTGGGCTAGCGAATTCGAGCTCGGTACCCGGGGATCCTCTAGA**AGGAGGgagta**ATGGCTCTAAATATCCTGTG
relE93-rev-lev-pst1	AAATCTTCTCTCATCCGCCAAAACAGCCAAGCTTGCATGCCTGCAGTCAATAATGACTGCGGATATTC

Primers used to clone into the pBAD33lev vector also include a RBS sequence (AGGAGG) and a spacer sequence (GAGTA) as indicated in bold.

### Toxicity and antitoxicity assays

Strains carrying the pBAD33-lev or its derivatives with the toxic genes and the pKK223-3-lev or its derivatives with the hypothetical antitoxin genes were diluted 10 times up to a dilution of 10^−6^ CFUs, and 10 μL was plated on minimum media supplied with casamino acids 0.2%, glycerol 1%, ampicillin, and Cm, and either glucose 1% or arabinose 1% and IPTG 0.01 mmol/L.

## Results

### Isolation of metagenomic DNA and construction of a library

**Figure 1:**
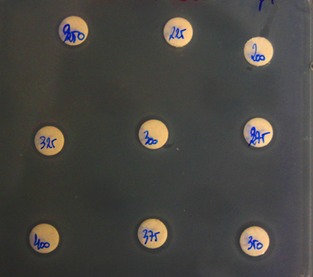
Growth of the Amp^R^ clone in the presence of different concentrations of ampicillin (Oxoid strips). Although some growth inhibition could be observed, there is regrowth of colonies inside the inhibition zone.

Metagenomic DNA was extracted from 2 L of filtered river water and was sheared before cloning in the fosmid vector pCC1fos (Epicenter®) as described in Material and Methods. Twenty colonies were picked and fosmid DNA extracted after a copy number amplification step. Fosmid DNAs were submitted to a restriction analysis and each clone provided a different pattern, which confirmed that the library is representative (results not shown). A total of 120,000 clones, each containing an insert of ∼35 kb, were pooled and screened for antibiotic resistance. A first screening of 12,500 clones was done on ampicillin-containing media (50 μg/mL), resulting in 20 resistant colonies (1/625 clones). DNA was isolated from these clones and analyzed by restriction, resulting in two different restriction patterns, suggesting the existence in the library of two types of Amp^R^ clones. One representative of each clone was selected and plated on media containing ceftazidime and piperacillin. One clone showed resistance to different classes of β-lactams (ampicillin, ceftazidime, and piperacillin) and shows a high MIC against ampicillin with many colonies growing in the inhibition zone, even at the highest concentration of antibiotic (Fig. 1).

### Identification of the gene(s) conferring a β-lactam resistance

**Figure 2:**
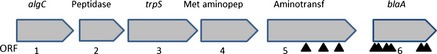
Genomic organization of ORFs 1–6: ORF1 (*algC*) encodes a phosphomannomutase, ORF2 a peptidase, ORF3 a tryptophanyl-tRNA synthetase, ORF4 a methionine aminopeptidase, ORF5 an aminotransferase, and ORF6 a periplasmic β-lactamase. The black triangles represent the transposon insertions resulting in the inactivation of the resistance to ampicillin.

**Figure 3:**
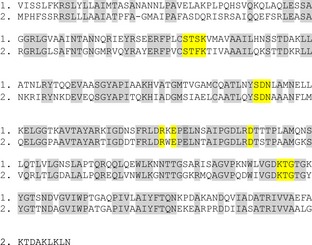
Alignment of the protein sequences corresponding to ORF6 encoded β-lactamase (1) and *Herminiimonas arsenoxydans* β-lactamase (2). The identical residues are highlighted in gray and the amino acids highlighted in yellow are those found in the class A β-lactamases as described in the results section.

To determine which gene(s) encode(s) the β-lactams resistance, in vitro transposon mutagenesis of the Amp^R^ clone was performed as described in Material and Methods. After transformation of *E. coli* cells, the clones were selected on plates containing Cm and kanamycin (to select for the fosmid- and transposon-containing bacteria). After replication on plates containing ampicillin, several Amp-sensitive clones were selected and further analyzed. The DNA sequence flanking the transposon was obtained using transposon-specific primers as described in the Material and Methods section. Three of the insertions causing the loss of resistance to ampicillin occurred in a gene open reading frames (ORF5), which encodes an aminotransferase, and the remaining six insertions were detected in ORF6, which codes for a β-lactamase (Fig. 2). The transposon insertions in ORF5 could have a polar effect on the expression of the β-lactamase gene (ORF6) downstream, explaining why they result in ampicillin sensitivity. Figure 2 shows the gene cluster comprising ORFs 1–6, whereas all ORFs present in the insert are described in Table S1 and presented in Figure S1. ORF6 is predicted to encode a periplasmic protein containing the Pfam 13,354 domain, which corresponds to the serine β-lactamase-like superfamily. The closest homolog is a β-lactamase found in *Herminiimonas arsenoxydans*, a β-proteobacterium (58% identity [169/291]) (Fig. 3).

**Figure 4:**
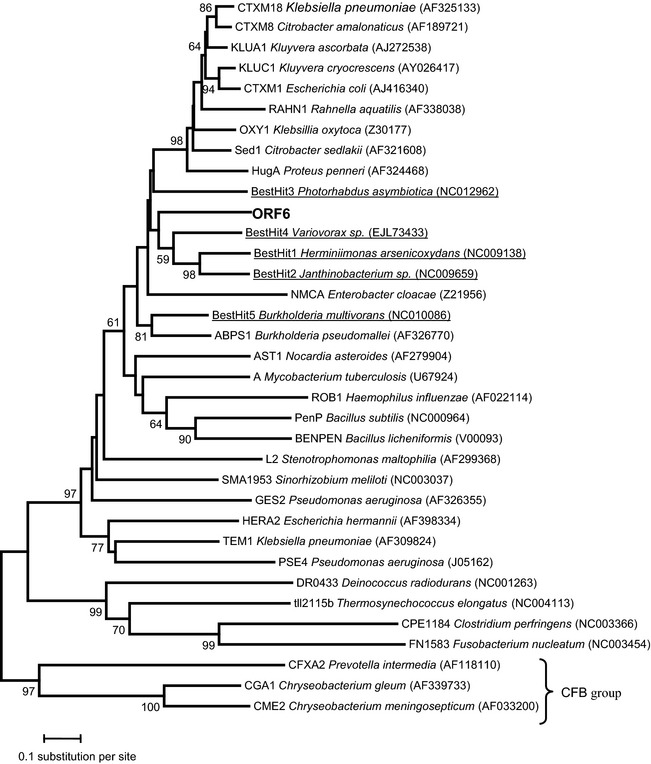
Phylogenetic tree of different class A β-lactamases (see text for details).

A phylogenetic tree shows that the β-lactamase encoded by ORF6 clusters with known class A β-lactamases enzymes, although presenting a different sequence (Fig. 4). The ORF6 encoded β-lactamase clusters with other similar class A enzymes present in the genomes of β-proteobacteria.

### Global analysis of the Amp^R^ clone

To gain information regarding the gene functions associated with the detected β-lactamase, the complete clone sequence was obtained (Genbank accession number KF033132). After aligning the fragments, a single contig of 47,977 nucleotides was obtained. Thirty-eight complete ORFs were detected, plus an incomplete ORF39 (Table S1 and Fig. S1). The first ORF encodes a phosphomannomutase (phosphoglucomutase), ORF2 encodes a peptidase, ORF3 a tryptophanyl-tRNA synthetase, ORF4 a methionine aminopeptidase, ORF5 an aminotransferase, and ORF6 the class A β-lactamase. ORFs 7–10 follow and are transcribed in the same forward orientation and they code for hypothetical proteins (Fig. S1). ORFs 11–14 are transcribed in the reverse orientation and correspond to the *kdpAFCB* genes which encode the components of a potassium transporting ATPase (Table S1 and Fig. S1). The ORFs 15 and 16, also transcribed in the reverse orientation, do not correspond to known proteins. Interestingly, ORF17, also transcribed in the reverse orientation, corresponds to the *hisC* gene encoding a histidinol phosphate aminotransferase involved in the biosynthesis of histidine. Other histidine biosynthesis genes are also found in the contig, namely, ORF21 (*hisD*) and ORFs 30–34 (*hisGBIF*), also transcribed in the reverse orientation. Between *hisC* and *hisD*, there are three ORFs (18–20) encoding, respectively, a RelA/SpoT protein (ORF18) and two outer membrane lipoproteins (ORFs 19 and 20). ORF22 encodes a ribosome-associated GTPase. ORFs 23–29 code for hypothetical proteins or proteins involved in toxin activity (ORFs 25 and 28) or in chromosome segregation (ORF27). ORF28 product is predicted to be a RelE toxin and ORF29 a ribbon–helix–helix protein CopG (see also section below). ORFs 34 and 35 are transcribed in the forward orientation and code for a C4-dicarboxylate transporter/tellurite resistance protein and a polyphosphate:nucleotide phosphotransferase, respectively. ORFs 36–39 are transcribed in the reverse orientation and code for a cobalamin S-adenosyl methionine transferase (ORF36) and different subunits of an ethanolamine transporter.

### ORFs 26 and 28 encode proteins that inhibit E. coli growth

**Figure 5:**
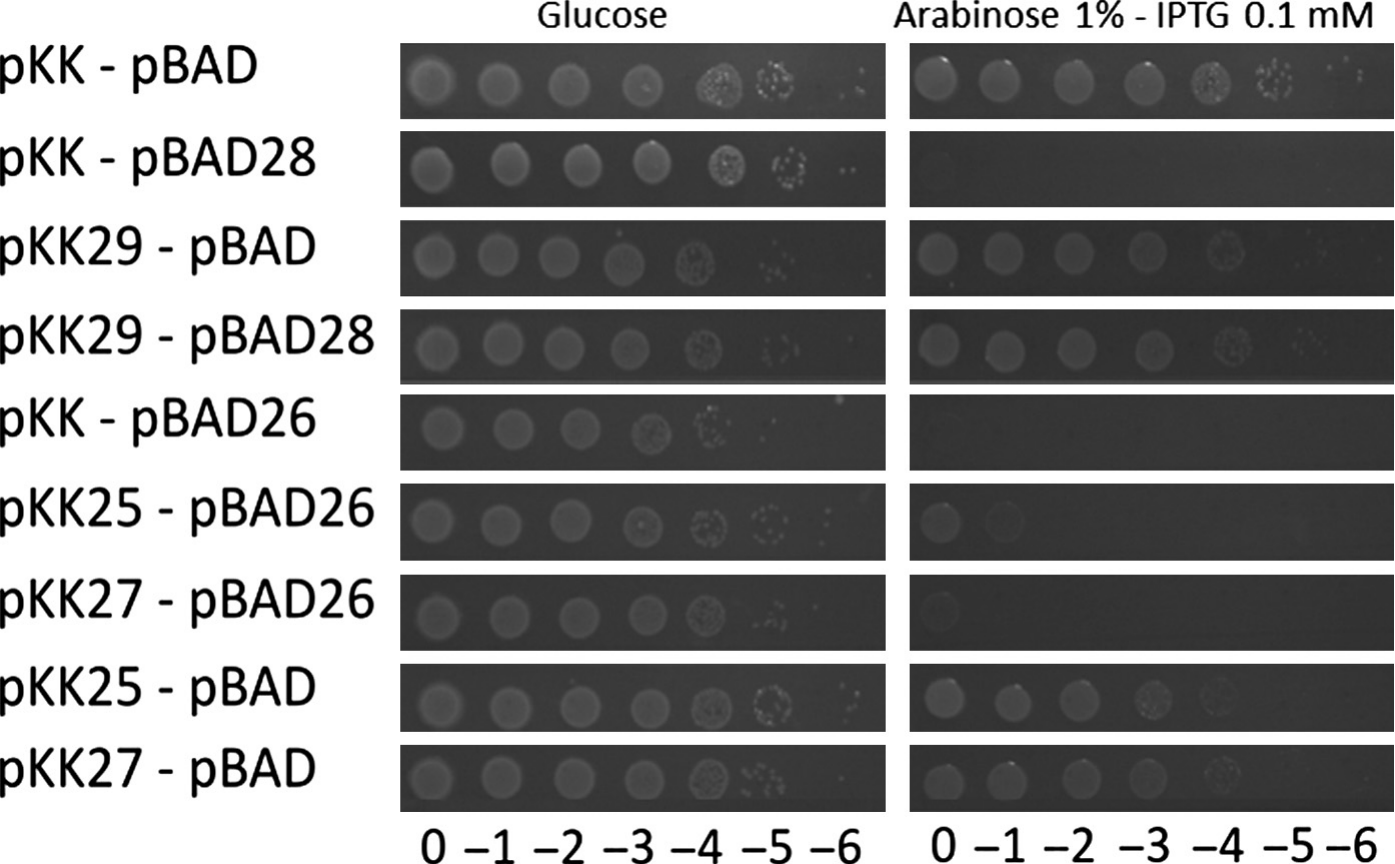
ORFs 28 and 29 constitute a RelBE toxin–antitoxin system, whereas ORF26 is a lone toxin. Serial dilutions of the DJ624Δ*ara* containing the pBAD33lev control vector or its derivatives with ORFs 24, 26, or *relE* (ORF28) and the control vector pKK223-3lev or its derivatives with ORF25, ORF27, or *relB* (ORF29) were plated on M9 minimal media containing arabinose (1%) and IPTG (0,1 mmol/L) and the appropriate antibiotics. Plates were incubated overnight at 37°C.

Two of the ORFs were predicted to encode type II toxins (Hayes and Van Melderen 2011). ORF25 was predicted to code for a toxin with a Fic/Doc domain found in Doc toxins (Engel et al. 2012) and ORF28 was predicted to encode a RelE-like toxin (Overgaard et al. 2009). Both toxins are translation inhibitors, although acting at different steps of translation. ORF29, flanking the predicted RelE toxin, encodes a predicted RelB antitoxin containing a RHH (**r**ibbon–**h**elix–**h**elix) domain. On the other hand, the predicted ORF25 Fic/Doc toxin is flanked by the small ORF24 and ORF26 with no conserved domains. To test whether ORF25 (Fic/Doc) and ORF28 (RelE) encode functional toxins, they were cloned and tested in *E. coli* using an inducible expression system as described in Material and Methods (Leplae et al. 2011). The potential toxic ORFs are cloned in an expression vector under the control of an arabinose-inducible promoter, which is repressed in the presence of glucose (Guzman et al. 1995). On the other hand, expression of the predicted antitoxins is driven by a pTac promoter, which is inducible by IPTG. Both plasmids, containing the toxin, and the antitoxin gene are compatible. The DJ624Δ*ara E. coli* strain was transformed by different combinations of construct and viability was assayed on minimal medium plates containing either glucose or arabinose and IPTG to induce expression of the different ORFs. Figure 5 shows that expression of ORF28 (RelE-like toxin) from the pBAD28 construct and the pKK control vector considerably reduces *E. coli* viability, whereas expression of ORF29 from the pKK29 has no effect on viability. However, coexpression of ORF28 and ORF29 restores the growth of *E. coli*. This result confirms that the ORF29–ORF28 system is indeed a type II toxin–antitoxin system. Expression of the predicted Doc toxin encoded by ORF25 from the pBAD did not show any toxicity (data not show). Interestingly, expression of ORF26 from the pBAD vector did inhibit *E. coli* growth. In an attempt to identify a potential antitoxin for the ORF26 toxin, the flanking ORFs (ORF25 and ORF27) were cloned in the pKK vector and their ability to restore viability was tested. Neither of these ORFs was, however, able to counteract ORF26-mediated toxicity. ORF24 was also tested and was not toxic (data not shown).

## Discussion

### Identification of an active class A β-lactamase

Our first aim was to detect, by functional screening, the expression of antibiotic resistance determinants coming from the environment, choosing river water, as it was hypothesized that the Zenne river in Brussels, being heavily polluted, could be a source of drug resistance genes (Baquero et al. 2008; Martinez et al. 2009; Allen et al. 2010; Garcia-Armisen et al. 2011). The Zenne being as well heavily polluted by the presence of heavy metals (Garcia-Armisen et al. 2011), it could also represent a source of both metal and antibiotic resistance genes (Baker-Austin et al. 2006). In a previous study, using the same water samples, we obtained 32 cultivable isolates showing resistance to at least one antibiotic among the nine tested (Cm, erythromycin, gentamicin, meropenem, aztreonam, tetracycline, amoxicillin, ofloxacin, and ceftazidime) (Garcia-Armisen et al. 2011). These cultivable bacteria belonged to different phyla (*Bacteroidetes* and *Proteobacteriales*) with a large representation of the *Pseudomonas* genus. However, a 16S clone library analysis revealed that there was no good correlation between the cultivable and the noncultivable bacteria as no *Pseudomonas* 16S sequence could be detected (Garcia-Armisen et al. 2011). This prompted us to look for novel resistance determinants by direct functional screening of a metagenomic library. The power of functional metagenomics for the discovery of novel antibiotic resistance genes has been previously established by the analysis of libraries of soil DNA (Allen et al. 2009; Lang et al. 2010; Torres-Cortes et al. 2011) and, more recently, from gulls (Martiny et al. 2011). Using this approach, new β-lactamase genes, different from the clinical enzymes, could be isolated from an Alaskan soil (Allen et al. 2009) and aminoglycoside resistance genes were discovered by screening a soil metagenomic library (Riesenfeld et al. 2004). Although gene expression using *E. coli* as host is not guaranteed (Uchiyama and Miyazaki 2009; Ekkers et al. 2012), our screening nevertheless resulted in the selection of a clone that is able to grow in the presence of high concentrations of β-lactam antibiotics. The β-lactamase encoded by ORF6 shows 74% similarity with a class A TEM β-lactamase from the β-proteobacterium *H. arsenoxydans* (Muller et al. 2007). The metagenomic β-lactamase clusters well with A-type enzymes (Fig. 4), suggesting that it belongs to this class of β-lactamase enzymes. Furthermore, our enzyme also contains conserved residues typical of class A enzymes in its active site (Majiduddin et al. 2002; Bos and Pleiss 2008). These residues, highlighted in Figure 3, are the following: a SXXK motif at positions 70–73, a SXN motif at about 130–132, a KT/SG motif at positions 234–236, and the **Ω** loop (Majiduddin et al. 2002; Bos and Pleiss 2008). The **Ω** loop (residues 161–179) is conserved in all class A β-lactamases and is directly involved in the catalytic reaction of the enzymes because it positions the general base Glu166. It also contains an Arg and an Asp separated by 15 residues, apart from each other, which stabilize the loop via the formation of a salt bridge, which are also present in our sequence (Bos and Pleiss 2008).

### *Identification of a* relBE *toxin–antitoxin system*

In this work we demonstrated that ORFs 28 and 29 correspond to a canonical *relBE* type II TA system. RelE toxin and RelB antitoxin have been described in *E. coli*, but were also identified in Archaea (Li et al. 2008, 2009; Overgaard et al. 2009; Shinohara et al. 2010). RelB, the antitoxin, has a ribbon–helix–helix structure and it represses the expression of the *relBE* operon using RelE as corepressor (Christensen et al. 2001; Christensen and Gerdes 2004; Overgaard et al. 2009). Interestingly, ORF18 codes for a protein with predicted RelA/SpoT activity. In *E. coli* and other bacteria, amino acid starvation triggers the stringent response, which is induced by the production of the alarmone ppGpp, synthesized by the RelA protein (Chatterji and Ojha 2001; Sharma and Chatterji 2010). The ppGpp molecules associate with the RNA polymerase to inhibit the transcription of stable ribosomal RNAs, resulting in lowered translational levels (Chatterji and Ojha 2001; Sharma and Chatterji 2010). At the same time, the stringent response also strongly induces the expression of the *relBE* locus, resulting in increased amounts of the mRNA translation inhibitor RelE, therefore coupling transcriptional (RelA) and translational (RelE) inhibition (Christensen et al. 2001; Christensen and Gerdes 2004). Surprisingly, expression of ORF25 containing a predicted FIC/Doc domain did not show any toxicity. The FIC domain catalyzes adenylylation and is found among others in virulence factors translocated into host cells by pathogenic bacteria and in Doc toxins. The consensus motif HXFX[(D/E)GNGRXXR was shown to be essential for the catalytic activity of Fic proteins (Engel et al. 2012), but this motif is degenerated in the putative toxin Doc encoded by ORF25, which leads to the hypothesis that this degenerated Doc lost its adenylylation activity. Indeed, the histidine residue of this motif was shown to be essential for Doc toxic activity (Magnuson and Yarmolinsky 1998; Garcia-Pino et al. 2008) as well as for FIC proteins (Engel et al. 2012). In ORF25, this residue is replaced by a glutamine (QFFFDGNKRTAR) which may explain why overexpression of ORF25 is not toxic despite its predicted Fic/Doc domain. In contrast, expression of ORF26 was shown to be toxic for *E. coli*. However, this toxin does not appear to belong to a classical type II system. Toxicity might be due to overproduction and not reflect any functional property.

### Histidine biosynthesis genes

Another striking feature of our sequence contig is the presence of different histidine biosynthesis genes, which are either clustered (*hisFIBG*) or separated by other ORFs (*hisC* and *hisD*). However, the gene coding for HisB, which catalyzes the sixth and the eight step of histidine biosynthesis pathway, is not present in our contig, meaning that the probability is high that we did not recover all the histidine biosynthesis genes in our fragment.

### From which bacterium is the insert coming from?

The sequenced insert presents several interesting features. First, the different ORFs do not show a clear phylogenetic affiliation. Eleven of the 39 predicted proteins have best hits that correspond to β-proteobacteria, nine to γ-proteobacteria, three to α-proteobacteria, four to δ-proteobacteria, and one to ε-proteobacteria. More intriguing is the fact that other phyla are represented as well among the best hits: ORF10 hypothetical protein best hit is with a *Calothrix* protein (Cyanobacteria), and ORF25 encoded protein shows the highest similarity with an *Acidobacteria* protein. The most surprising result of the Blast analysis concerns ORF35 encoding a polyphosphate:nucleotide phosphotransferase which shows the highest similarity with a protein coming from a methanogenic Archeon (Euryarcheota). The histidine biosynthesis genes appear also to have homologs in quite different bacteria (α-proteobacteria for HisC and HisG, γ-proteobacteria for HisD and HisF, a β-proteobacterium for HisB, and a bacterium belonging to the Bacteroidetes for HisI).

## Supporting Information

Figure S1 Genomic organization of the 39 ORFs present in the insert of the Amp^R^ clone.

Table S1 The complete list of the 39 ORFs detected in the insert of the AmpR clone.

## References

[b1] Allen HK, Moe LA, Rodbumrer J, Gaarder A, Handelsman J (2009). Functional metagenomics reveals diverse beta-lactamases in a remote Alaskan soil. ISME J.

[b2] Allen HK, Donato J, Wang HH, Cloud-Hansen KA, Davies J, Handelsman J (2010). Call of the wild: antibiotic resistance genes in natural environments. Nat. Rev. Microbiol.

[b3] Andam CP, Fournier GP, Gogarten JP (2011). Multilevel populations and the evolution of antibiotic resistance through horizontal gene transfer. FEMS Microbiol. Rev.

[b4] Baker-Austin C, Wright MS, Stepanauskas R, McArthur JV (2006). Co-selection of antibiotic and metal resistance. Trends Microbiol.

[b5] Baquero F, Martinez JL, Canton R (2008). Antibiotics and antibiotic resistance in water environments. Curr. Opin. Biotechnol.

[b6] Bonneaud N, Ozier-Kalogeropoulos O, Li GY, Labouesse M, Minvielle-Sebastia L, Lacroute F (1991). A family of low and high copy replicative, integrative and single-stranded *S. cerevisiae**E. coli* shuttle vectors. Yeast.

[b7] Bos F, Pleiss J (2008). Conserved water molecules stabilize the Omega-loop in class A beta-lactamases. Antimicrob. Agents Chemother.

[b8] Brosius J, Holy A (1984). Regulation of ribosomal RNA promoters with a synthetic lac operator. Proc. Natl Acad. Sci. USA.

[b9] Calomiris JJ, Armstrong JL, Seidler RJ (1984). Association of metal tolerance with multiple antibiotic resistance of bacteria isolated from drinking water. Appl. Environ. Microbiol.

[b10] Casadaban MJ, Cohen SN (1980). Analysis of gene control signals by DNA fusion and cloning in *Escherichia coli*. J. Mol. Biol.

[b11] Chatterji D, Ojha AK (2001). Revisiting the stringent response, ppGpp and starvation signaling. Curr. Opin. Microbiol.

[b12] Christensen SK, Gerdes K (2004). Delayed-relaxed response explained by hyperactivation of RelE. Mol. Microbiol.

[b13] Christensen SK, Mikkelsen M, Pedersen K, Gerdes K (2001). RelE, a global inhibitor of translation, is activated during nutritional stress. Proc. Natl. Acad. Sci. USA.

[b14] de la Cruz F, Davies J (2000). Horizontal gene transfer and the origin of species: lessons from bacteria. Trends Microbiol.

[b15] Ekkers DM, Cretoiu MS, Kielak AM, Elsas JD (2012). The great screen anomaly: a new frontier in product discovery through functional metagenomics. Appl. Microbiol. Biotechnol.

[b16] Engel P, Goepfert A, Stanger FV, Harms A, Schmidt A, Schirmer T (2012). Adenylylation control by intra- or intermolecular active-site obstruction in Fic proteins. Nature.

[b17] Garcia-Armisen T, Vercammen K, Passerat J, Triest D, Servais P, Cornelis P (2011). Antimicrobial resistance of heterotrophic bacteria in sewage-contaminated rivers. Water Res.

[b18] Garcia-Pino A, Christensen-Dalsgaard M, Wyns L, Yarmolinsky M, Magnuson RD, Gerdes K (2008). Doc of prophage P1 is inhibited by its antitoxin partner Phd through fold complementation. J. Biol. Chem.

[b19] Gietz RD, Schiestl RH (2007). Quick and easy yeast transformation using the LiAc/SS carrier DNA/PEG method. Nat. Protoc.

[b20] Gootz TD (2010). The global problem of antibiotic resistance. Crit. Rev. Immunol.

[b21] Gouy M, Guindon S, Gascuel O (2010). SeaView version 4: a multiplatform graphical user interface for sequence alignment and phylogenetic tree building. Mol. Biol. Evol.

[b22] Grass G, Rensing L, Rensing C (2011). Metal toxicity. Metallomics.

[b23] Guzman LM, Belin D, Carson MJ, Beckwith J (1995). Tight regulation, modulation, and high-level expression by vectors containing the arabinose PBAD promoter. J. Bacteriol.

[b24] Hall BG, Barlow M (2004). Evolution of the serine beta-lactamases: past, present and future. Drug Resist. Updat.

[b25] Hayes F, Van Melderen L (2011). Toxins-antitoxins: diversity, evolution and function. Crit. Rev. Biochem. Mol. Biol.

[b26] Kim EH, Nies DH, McEvoy MM, Rensing C (2011). Switch or funnel: how RND-type transport systems control periplasmic metal homeostasis. J. Bacteriol.

[b27] Knapp CW, McCluskey SM, Singh BK, Campbell CD, Hudson G, Graham DW (2011). Antibiotic resistance gene abundances correlate with metal and geochemical conditions in archived Scottish soils. PLoS ONE.

[b28] Kohanski MA, DePristo MA, Collins JJ (2010). Sublethal antibiotic treatment leads to multidrug resistance via radical-induced mutagenesis. Mol. Cell.

[b29] Lang KS, Anderson JM, Schwarz S, Williamson L, Handelsman J, Singer RS (2010). Novel florfenicol and chloramphenicol resistance gene discovered in Alaskan soil by using functional metagenomics. Appl. Environ. Microbiol.

[b30] Larkin MA, Blackshields G, Brown NP, Chenna R, McGettigan PA, McWilliam H (2007). Clustal W and Clustal X version 2.0. Bioinformatics.

[b31] Leplae R, Geeraerts D, Hallez R, Guglielmini J, Dreze P, Van Melderen L (2011). Diversity of bacterial type II toxin-antitoxin systems: a comprehensive search and functional analysis of novel families. Nucleic Acids Res.

[b32] Li GY, Zhang Y, Inouye M, Ikura M (2008). Structural mechanism of transcriptional autorepression of the *Escherichia coli* RelB/RelE antitoxin/toxin module. J. Mol. Biol.

[b33] Li GY, Zhang Y, Inouye M, Ikura M (2009). Inhibitory mechanism of *Escherichia coli* RelE-RelB toxin-antitoxin module involves a helix displacement near an mRNA interferase active site. J. Biol. Chem.

[b34] Lloyd DR, Phillips DH (1999). Oxidative DNA damage mediated by copper(II), iron(II) and nickel(II) fenton reactions: evidence for site-specific mechanisms in the formation of double-strand breaks, 8-hydroxydeoxyguanosine and putative intrastrand cross-links. Mutat. Res.

[b35] Magnuson R, Yarmolinsky MB (1998). Corepression of the P1 addiction operon by Phd and Doc. J. Bacteriol.

[b36] Majiduddin FK, Materon IC, Palzkill TG (2002). Molecular analysis of beta-lactamase structure and function. Int. J. Med. Microbiol.

[b37] Martinez JL, Rojo F (2011). Metabolic regulation of antibiotic resistance. FEMS Microbiol. Rev.

[b38] Martinez JL, Fajardo A, Garmendia L, Hernandez A, Linares JF, Martinez-Solano L (2009). A global view of antibiotic resistance. FEMS Microbiol. Rev.

[b39] Martiny AC, Martiny JB, Weihe C, Field A, Ellis JC (2011). Functional metagenomics reveals previously unrecognized diversity of antibiotic resistance genes in gulls. Front. Microbiol.

[b40] Muller D, Medigue C, Koechler S, Barbe V, Barakat M, Talla E (2007). A tale of two oxidation states: bacterial colonization of arsenic-rich environments. PLoS Genet.

[b41] Overgaard M, Borch J, Gerdes K (2009). RelB and RelE of *Escherichia coli* form a tight complex that represses transcription via the ribbon-helix-helix motif in RelB. J. Mol. Biol.

[b42] Riesenfeld CS, Goodman RM, Handelsman J (2004). Uncultured soil bacteria are a reservoir of new antibiotic resistance genes. Environ. Microbiol.

[b43] Sharma UK, Chatterji D (2010). Transcriptional switching in *Escherichia coli* during stress and starvation by modulation of sigma activity. FEMS Microbiol. Rev.

[b44] Shinohara M, Guo JX, Mori M, Nakashima T, Takagi H, Nishimoto E (2010). The structural mechanism of the inhibition of archaeal RelE toxin by its cognate RelB antitoxin. Biochem. Biophys. Res. Commun.

[b45] Talavera G, Castresana J (2007). Improvement of phylogenies after removing divergent and ambiguously aligned blocks from protein sequence alignments. Syst. Biol.

[b46] Tamura K, Peterson D, Peterson N, Stecher G, Nei M, Kumar S (2011). MEGA5: molecular evolutionary genetics analysis using maximum likelihood, evolutionary distance, and maximum parsimony methods. Mol. Biol. Evol.

[b47] Torres-Cortes G, Millan V, Ramirez-Saad HC, Nisa-Martinez R, Toro N, Martinez-Abarca F (2011). Characterization of novel antibiotic resistance genes identified by functional metagenomics on soil samples. Environ. Microbiol.

[b48] Uchiyama T, Miyazaki K (2009). Functional metagenomics for enzyme discovery: challenges to efficient screening. Curr. Opin. Biotechnol.

[b49] Xiao H, Kalman M, Ikehara K, Zemel S, Glaser G, Cashel M (1991). Residual guanosine 3′,5′-bispyrophosphate synthetic activity of *relA* null mutants can be eliminated by *spoT* null mutations. J. Biol. Chem.

[b50] Zhang T, Zhang XX, Ye L (2011). Plasmid metagenome reveals high levels of antibiotic resistance genes and mobile genetic elements in activated sludge. PLoS ONE.

