# Supplementary figures and images for: Identification of a metagenomic gene cluster containing a new class A beta-lactamase and toxin-antitoxin systems
Source: Microbiologyopen. 2013 Jul 22;2(4):674–83. doi: 10.1002/mbo3.104 (PMC3948609; doi:10.1002/mbo3.104)

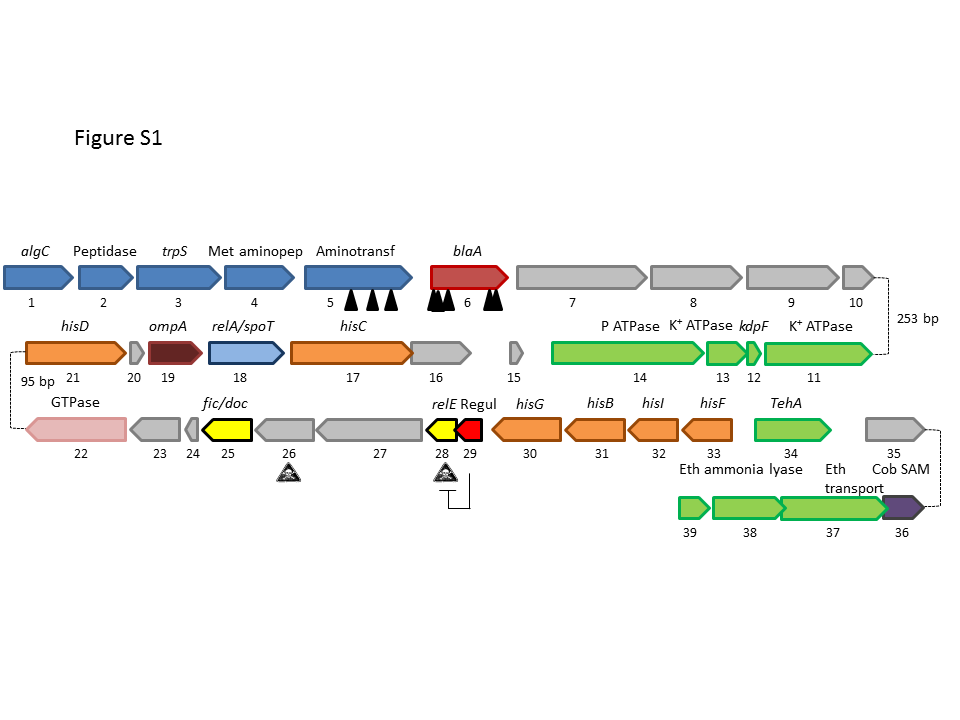

Supplement: Figure S1 — Genomic organization of the 39 ORFs present in the insert of the AmpR clone. [file mbo30002-0674-sd1.tif]
