# Supplementary material for: Identification of a metagenomic gene cluster containing a new class A beta-lactamase and toxin-antitoxin systems
Source: Microbiologyopen. 2013 Jul 22;2(4):674–83. doi: 10.1002/mbo3.104 (PMC3948609; doi:10.1002/mbo3.104)
Supplement: Table S1 — The complete list of the 39 ORFs detected in the insert of the AmpR clone. [file mbo30002-0674-sd2.docx]

| **ORF** | **Position** | **Orientation** | **Aas** | **Signal** | **Best hit** | **% simil.** | **Gene** | **Function** | **Organism** | **Phylum** |
| --- | --- | --- | --- | --- | --- | --- | --- | --- | --- | --- |
| 1 | 3-911 | Forward | 302 | No | ref\|NP_901842.1\| | 86% |  | Phosphomannomutase | *Chromobacterium violaceum (β proteo)* | β-proteobacteria |
| 2 | 1002-1661 | Forward | 219 | No | [ref\|YP_002796096.1\|](http://www.ncbi.nlm.nih.gov/protein/226941022?report=genbank&log$=protalign&blast_rank=1&RID=RX45EM5V014) | 74% |  | Peptidase M 50 | *Laribacter Hongkonggensis (β proteo)* | β-proteobacteria |
| 3 | 1787 -2869 | Forward | 360 | No | [ref\|YP_413000.1\|](http://www.ncbi.nlm.nih.gov/protein/82703434?report=genbank&log$=protalign&blast_rank=1&RID=RX4JUN7G01R) | 73% | *trpS* | Trp aminoacyl-tRNA synthetase | *Nitrosospira multiformis (β proteo)* | β-proteobacteria |
| 4 | 2879-3682 | Forward | 267 | No | [ref\|NP_902067.1\|](http://www.ncbi.nlm.nih.gov/protein/34497852?report=genbank&log$=protalign&blast_rank=1&RID=RX4TYR1P014) | 82% |  | Met aminopeptidase | *Holophaga foetida (δ proteo)* | β-proteobacteria |
| 5 | 3830-5218 | Forward | 462 | No | [ref\|ZP_09577385.1\|](http://www.ncbi.nlm.nih.gov/protein/373486713?report=genbank&log$=protalign&blast_rank=1&RID=RX4ZBAP3014) | 69% |  | Aminotransferase | *Anaeromyxobacter sp. (δ proteo)* | δ-proteobacteria |
| 6 | 5645-6574 | Forward | 309 | Yes | [ref\|YP_001098933.1\|](http://www.ncbi.nlm.nih.gov/protein/134093858?report=genbank&log$=protalign&blast_rank=1&RID=RX5604T6014) | 74% | *blaA* | Beta-lactamase | *Herminiimonas arsenooxydans (β proteo)* | β-proteobacteria |
| 7 | 6769-8655 | Forward | 628 | No | [gb\|EKD41383.1\|](http://www.ncbi.nlm.nih.gov/protein/406897421?report=genbank&log$=protalign&blast_rank=1&RID=RX5BB8S701R) | 43% | *lenF* | Hypothetical: Ca2+ binding | Unknow *(γ proteo)* | γ-proteobacteria |
| 8 | 8747-10621 | Forward | 624 | No | [gb\|EED35397.1\|](http://www.ncbi.nlm.nih.gov/protein/219679048?report=genbank&log$=protalign&blast_rank=1&RID=B00JXB38016) | 44% | *lenF* | Hypothetical: Ca2+ binding | Unknow *(γ proteo)* | γ-proteobacteria |
| 9 | 10813-12003 | Forward | 396 | No | No Hit |  |  |  |  |  |
| 10 | 12049-12363 | Forward | 104 | No | [ref\|YP_007135507.1\|](http://www.ncbi.nlm.nih.gov/protein/428297201?report=genbank&log$=protalign&blast_rank=1&RID=RX5XBYZW015) | 81% |  | Antibiotic biosynthesis protein | *Calothrix sp. (Cyano)* | Firmicutes |
| 11 | 12616-14364 | Reverse | 582 | No | [ref\|YP_003675025.1\|](http://www.ncbi.nlm.nih.gov/protein/297539256?report=genbank&log$=protalign&blast_rank=1&RID=RZ1GMC3R014) | 78% | *kdpA* | K-transporting ATPase subunit A | *Methylotenera versatilis (β proteo)* | β-proteobacteria |
| 12 | 14507-14596 | Reverse | 29 | No | No Hit |  |  |  |  |  |
| 13 | 14596-15183 | Reverse | 195 | No | [ref\|ZP_10571048.1\|](http://www.ncbi.nlm.nih.gov/protein/398812279?report=genbank&log$=protalign&blast_rank=1&RID=RZ21GN7F014) | 72% | *kdpC* | K-transporting ATPase subunit C | *Variovorax (β proteo)* | β-proteobacteria |
| 14 | 15194-17266 | Reverse | 690 | No | [emb\|CCA85765.1\|](http://www.ncbi.nlm.nih.gov/protein/344173095?report=genbank&log$=protalign&blast_rank=1&RID=B01G7N1V013) | 83% | *kdpB* | K-transporting ATPase subunit B | *Ralstonia syzygii (β proteo)* | β-proteobacteria |
| 15 | 17745-17876 | Reverse | 43 | No | No hit |  |  |  |  |  |
| 16 | 18421-19122 | Reverse | 233 | No | No hit |  |  |  |  |  |
| 17 | 19119-20771 | Reverse | 550 | No | [ref\|YP_003550769.1\|](http://www.ncbi.nlm.nih.gov/protein/294084012?report=genbank&log$=protalign&blast_rank=1&RID=RZ2YHUUK015) | 63% | *hisC* | Histidinol phosphate aminotransferase | *Candidatus Puniceispirillum marinum (α proteo)* | α-proteobacteria |
| 18 | 20870-21859 | Reverse | 329 | No | [ref\|ZP_03247428.1\|](http://www.ncbi.nlm.nih.gov/protein/208780085?report=genbank&log$=protalign&blast_rank=1&RID=RZ34W2YR01R) | 65% |  | RelA/SpoT | *Francisella novicida (γ proteo)* | γ-proteobacteria |
| 19 | 21968-22543 | Reverse | 191 | Yes (Lpp) | [ref\|YP_001155566.1\|](http://www.ncbi.nlm.nih.gov/protein/145588969?report=genbank&log$=protalign&blast_rank=1&RID=RZ39ZFNK014) | 59% |  | OmpA/MotB domain | *Polynucleobacter necessarius (β proteo)* | β-proteobacteria |
| 20 | 22613-22756 | Reverse | 47 | Yes (Lpp) | [ref\|YP_001156092.1\|](http://www.ncbi.nlm.nih.gov/protein/145589495?report=genbank&log$=protalign&blast_rank=1&RID=RZ3G7SPK014) | 70% |  | Lipoprotein | *Polynucleobacter necessarius (β proteo)* | β-proteobacteria |
| 21 | 22797-24092 | Reverse | 431 | No | ref\|YP_004647704.1\| | 72% | *hisD* | Histidinol dehydrogenase | *Francisella novicida (γ proteo)* | γ-proteobacteria |
| 22 | 24191-25258 | Reverse | 355 | No | [gb\|EKD25703.1\|](http://www.ncbi.nlm.nih.gov/protein/406876015?report=genbank&log$=protalign&blast_rank=1&RID=RZ41B1K9014) | 81% |  | Ribosome-associated GTPase | *Uncultured bacterium* | Flavobacteria |
| 23 | 25285-25926 | Reverse | 213 | No | [ref\|YP_825218.1\|](http://www.ncbi.nlm.nih.gov/protein/116623062?report=genbank&log$=protalign&blast_rank=1&RID=RZ49ZP8X01R) | 55% |  | Hypothetical protein | *Candidatus Solibacter usitatus (Acidobacteria)* | Acidobacteria |
| 24 | 26066-26176 | Reverse | 36 | No | [ref\|YP_341468.1\|](http://www.ncbi.nlm.nih.gov/protein/77361893?report=genbank&log$=protalign&blast_rank=1&RID=RZ4GHD4B014) | 78% |  | Hypothetical protein | *Pseudoalteromonas haloplanktis (γ proteo)* |  |
| 25 | 26189-26875 | Reverse | 228 | No | [ref\|ZP_09754609.1\|](http://www.ncbi.nlm.nih.gov/protein/375108352?report=genbank&log$=protalign&blast_rank=1&RID=RZ4PB2F4015) | 65% |  | Fic/Doc family of toxins | *Alishewanella sheotgali (γ proteo)* | γ-proteobacteria |
| 26 | 26880-27686 | Reverse | 268 | No | [ref\|YP_001490309.1\|](http://www.ncbi.nlm.nih.gov/protein/157737626?report=genbank&log$=protalign&blast_rank=1&RID=RZ4X0PVK014) | 75% |  | Toxin activity (experimental) | *Arcobacter butzleri (ε proteo)* | ε-proteobacteria |
| 27 | 27701-29062 | Reverse | 453 | No | [ref\|YP_003052127.1\|](http://www.ncbi.nlm.nih.gov/protein/254000064?report=genbank&log$=protalign&blast_rank=1&RID=RZ51658G015) | 51% |  | Hypothetical protein: chromosome segregation | *Methylovorus glucosetrophus (β proteo)* | β-proteobacteria |
| 28 | 29150-29443 | Reverse | 97 | No | [ref\|YP_007326874.1\|](http://www.ncbi.nlm.nih.gov/protein/436842496?report=genbank&log$=protalign&blast_rank=1&RID=RZ563ACD014) | 62% |  | RelE toxin (experimental) | *Desulfovibrio hydrothermalis (δ proteo)* | Acidobacteria |
| 29 | 29434-29673 | Reverse | 79 | No | [ref\|ZP_13490993.1\|](http://www.ncbi.nlm.nih.gov/protein/418937344?report=genbank&log$=protalign&blast_rank=1&RID=RZ5BTTEF014) | 67% |  | Ribbon-helix-helix protein CopG antitoxin (experimental) | *Rhizobium sp. (α proteo)* | γ-proteobacteria |
| 30 | 29837-30721 | Reverse | 294 | No | [ref\|ZP_00951686.1\|](http://www.ncbi.nlm.nih.gov/protein/83858164?report=genbank&log$=protalign&blast_rank=1&RID=RZ5G6JYP01R) | 68% | *hisG* | ATP phosphoribosyl transferase | *Oceanicaulis sp. (α proteo)* | α-proteobacteria |
| 31 | 30759-31544 | Reverse | 261 | No | [ref\|YP_003982489.1\|](http://www.ncbi.nlm.nih.gov/protein/311109636?report=genbank&log$=protalign&blast_rank=1&RID=RZ5M11TZ015) | 66% | *hisB* | Histidinol phosphate phosphatase | *Achromobacter xyloxydans (β proteo)* | β-proteobacteria |
| 32 | 31563-32192 | Reverse | 209 | No | [ref\|ZP_09630724.1\|](http://www.ncbi.nlm.nih.gov/protein/374373064?report=genbank&log$=protalign&blast_rank=1&RID=RZ5RYDMD014) | 75% | *hisI* | Phosphoribosyl hydrolase | *Niabella soli (Bacteroidetes)* | bacteroidetes |
| 33 | 32262-33029 | Reverse | 255 | No | [ref\|ZP_04978382.1\|](http://www.ncbi.nlm.nih.gov/protein/254362269?report=genbank&log$=protalign&blast_rank=1&RID=RZ5WPVB2015) | 78% | *hisF* | Imidazoleglycerol phosphate synthase | *Mannheimia hemolytica (γ proteo)* | γ-proteobacteria |
| 34 | 33370-34317 | Forward | 315 | No | [gb\|EEO36588.1\|](http://www.ncbi.nlm.nih.gov/protein/229421541?report=genbank&log$=protalign&blast_rank=2&RID=B0C5H11B016) | 69% | *tehA* | C4-dicarboxylate transporter/tellurite resistance | *Fusobacterium mortiferum (Bacteroidetes)* | Fusobacteria |
| 35 | 34919-35842 | Forward | 307 | No | [ref\|YP_001403276.1\|](http://www.ncbi.nlm.nih.gov/protein/154149658?report=genbank&log$=protalign&blast_rank=1&RID=RZ680FR5015) | 69% |  | polyphosphate:nucleotide phosphotransferase | *Methanoregula boonei (Archaea-Euryarcheota)* | Euryarcheota |
| 36 | 35926-36456 | Reverse | 176 | No | [ref\|YP_003956191.1\|](http://www.ncbi.nlm.nih.gov/protein/310823833?report=genbank&log$=protalign&blast_rank=1&RID=RZ6C08MR015) | 61% |  | Cobalamin adenosyl transferase | *Stigmatella aurantiaca (δ proteo)* | δ-proteobacteria |
| 37 | 36440-37765 | Reverse | 441 | No | [ref\|ZP_09972145.1\|](http://www.ncbi.nlm.nih.gov/protein/383816754?report=genbank&log$=protalign&blast_rank=1&RID=RZ6FD0R8015) | 60% |  | Ethanolamine transporter | *Serratia sp. (γ proteo)* | β-proteobacteria |
| 38 | 37775-38600 | Reverse | 281 | No | [ref\|ZP_05826853.1\|](http://www.ncbi.nlm.nih.gov/protein/260554632?report=genbank&log$=protalign&blast_rank=1&RID=RZ6RJ52B015) | 62% |  | Ethanolamine ammonia lyase light subunit | *Acinetobacter baumanii (γ proteo)* | γ-proteobacteria |
| 39 | 40320-40616 | Reverse | 98 | No | [ref\|YP_521868.1\|](http://www.ncbi.nlm.nih.gov/protein/89899397?report=genbank&log$=protalign&blast_rank=1&RID=RZGJ7KDH01R) | 96% |  | Ethanolamine ammonia lyase heavy subunit (incomplete) | *Rhodoferax ferrireducens (β proteo)* | β-proteobacteria |
